# Spinal epidural hematoma following a thoracic epidural in a child with sickle cell disease

**DOI:** 10.1002/ccr3.1016

**Published:** 2017-05-31

**Authors:** Madhankumar Sathyamoorthy, Benjamin Walker, Melissa M. Rhodes, Ike Eriator

**Affiliations:** ^1^ Department of AnesthesiologyUniversity of Mississippi Medical CenterJacksonMississippi39216USA; ^2^ Department of AnesthesiologyUniversity of Wisconsin School of Medicine and Public HealthAmerican Family Children's HospitalMadisonWisconsin53792USA; ^3^ Department of PediatricsUniversity of Mississippi Medical CenterJacksonMississippi39216USA

**Keywords:** Pediatric, sickle cell disease, spinal epidural hematoma, thoracic epidural

## Abstract

Acute liver dysfunction in the perioperative period may increase the risk of epidural hematoma in a patient with a neuraxial catheter. Coagulation testing needs to be carefully monitored in these patients. An epidural hematoma should be ruled out urgently by CT or MRI in cases of a persistent motor block.

## Background

A twelve‐year‐old child with sickle cell disease had a thoracic epidural catheter for an open common bile duct exploration. On the first postoperative day, he developed motor paralysis in his right leg caused by a spinal hematoma from T7 to T9. He had a decompressive laminectomy with complete recovery in 6 months.

Spinal epidural hematoma (EH) following a neuraxial block is a rare complication. Its true incidence in children is unknown. The rate in adult patients varies from 1 in 3352 in cardiac surgery 1 to less than 1 in 150,000 in labor epidurals 2. Most reported cases of epidural hematoma have been in adult patients taking anticoagulants or after traumatic catheter placements 3. EH was not reported after thousands of neuraxial catheters in children from observational studies and audits 4, 5, 6. To our knowledge, this is the first reported case of an epidural hematoma associated with a thoracic epidural catheter in a child with sickle cell disease.

## Case Report

A 12‐year‐old, 48‐kg male with sickle cell disease (SCD) was admitted for right upper quadrant pain, exacerbation of anemia (Hgb 5.8 g∙dL^−1^; baseline 7.1 g∙dL^−1^), and extreme hyperbilirubinemia (total bilirubin 62.58 mg∙dL^−1^, direct bilirubin > 20 mg∙dL^−1^). Abdominal ultrasound was consistent with choledocholithiasis. His liver enzymes were within normal limits with alanine aminotransferase (ALT) at 27 U/L (ref: 10‐55 U/L) and aspartate aminotransferase (AST) at 96 U/L (ref: 15‐40 U/L). The coagulation testing showed prothrombin time (PT) of 13.5 sec (ref: 9.4‐12.5 sec), an international normalized ratio (INR) of 1.21 (ref: 0.8‐1.1), and activated partial thromboplastin time (aPTT) of 28.8 sec (25.1‐36.5 sec). He had an unsuccessful endoscopic retrograde cholangiography (ERCP) complicated by mild pancreatitis. After 8 days of bowel rest, antibiotics, and total parenteral nutrition (TPN), he was scheduled for open common bile duct exploration in the operating room. He had episodes of sickle cell crises and acute chest syndrome in the past but none in the last 3 years. His past surgical history included laparoscopic cholecystectomy under general anesthesia without any complications. His medications at home included folic acid and hydroxyurea. He had received four units of packed red blood cells (PRBC) earlier in the hospital course and one unit of PRBC the day before the scheduled procedure to treat anemia. His laboratory tests on the day of surgery included Hgb of 9.9 g∙dL^−1^ and platelet count of 350 × 10^9^/L.

**Figure 1:**
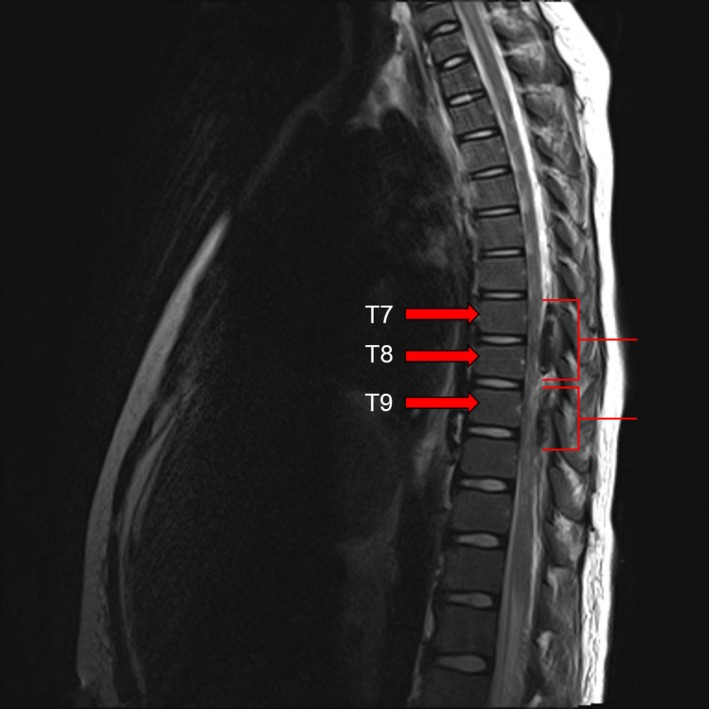
MRI T2 sagittal image is showing a mixed predominately hypo‐intense lesion within the spinal canal spanning T7‐T10.

On the day of the procedure, he was icteric and had stable vital signs with an oxygen saturation of 95% on room air. Following a team discussion of the risks and benefits of the anesthetic options, a general anesthesia with an asleep placement of a thoracic epidural catheter was chosen. Following endotracheal intubation, a thoracic epidural catheter was inserted at the level of T9‐T10 on the first attempt with a paramedian approach and loss of resistance to saline technique. A 20‐gauge multi‐orifice classic nylon closed‐tip epidural catheter (Perifix^®^ Braun Medical Inc. Bethlehem, PA.) was threaded 5 cm into the epidural space and secured at 9 cm at the skin. A test dose of 3 mL of 1.5% lidocaine with 1:200,000 epinephrine and aspiration of the catheter were both negative for intravascular catheter placement. The catheter was bolused with 0.5% ropivacaine in divided doses for a total of 6 mL. The procedure lasted 3.5 h. He lost approximately 500 mL of blood mainly from diffuse oozing from the surgical site. Two units of packed red blood cells were transfused to maintain Hb level above 10 g∙dL^−1^, and 3 L of lactated Ringer's solution was given. During the procedure, his esophageal core temperature was between 36.6 and 38.1°C, urine output was more than 2 mL∙kg h^−1^, and he remained hemodynamically stable. He was extubated at the end of the procedure with stable vital signs. A continuous infusion with 0.2% ropivacaine and fentanyl 2 mcg∙mL^−1^ at 6 mL h^−1^was started during the procedure and continued postoperatively. In the postanesthesia care unit (PACU), he was comfortable with sensory block up to T4 and without any motor block in his legs. Immediate postoperative laboratory tests showed Hgb of 11.9 g∙dL‐1, platelet count of 266 × 10^9^/L, INR of 1.41, aPTT of 33.7 sec, ALT of 635 U/L, and AST of 2058 U/L.

**Figure 2:**
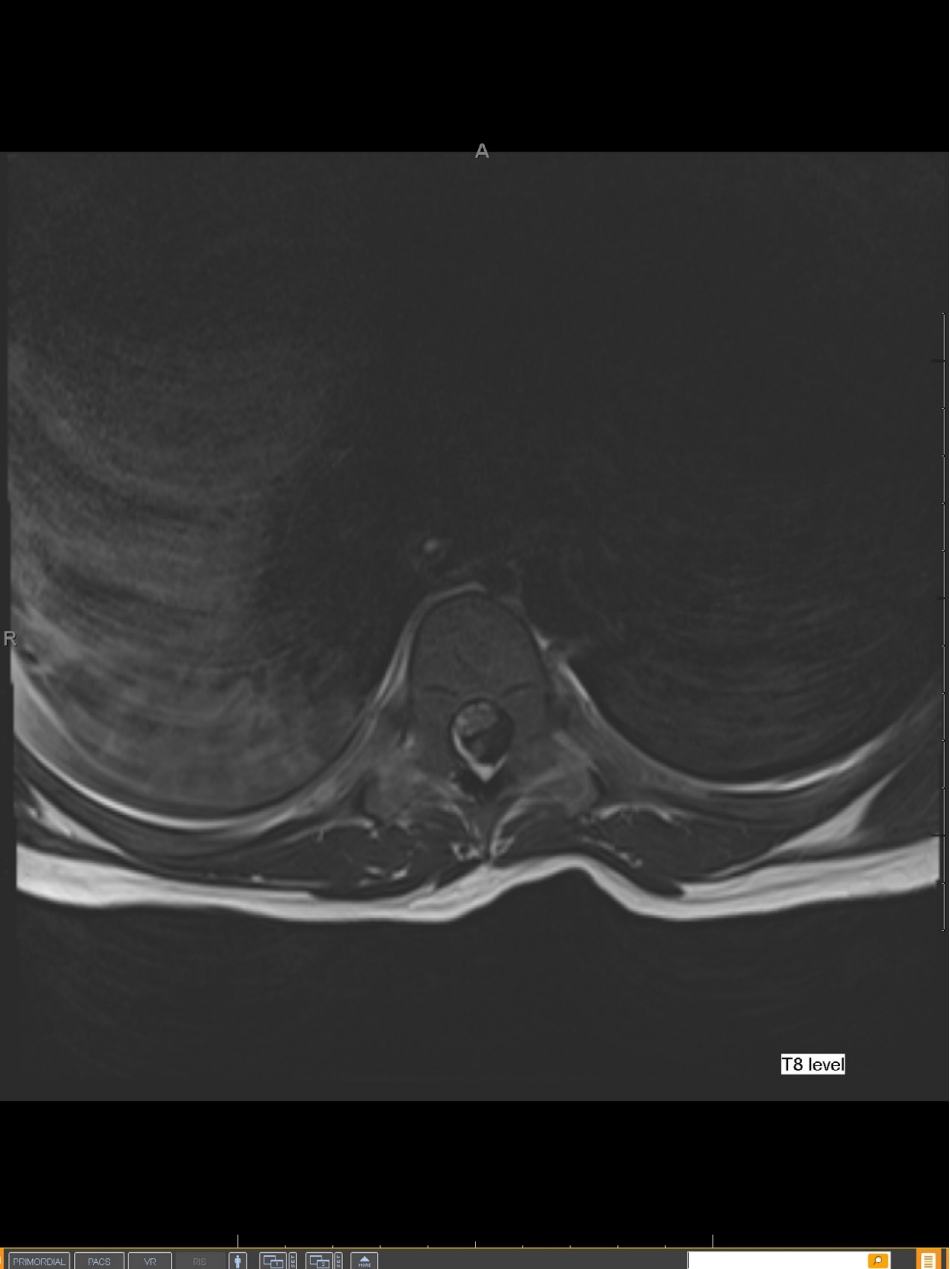
MRI axial image is showing a predominately T2 hypo‐intense lesion within the thoracic spinal canal at T8 level compressing the cord anteriorly and increased cord signal consistent with edema.

**Figure 3:**
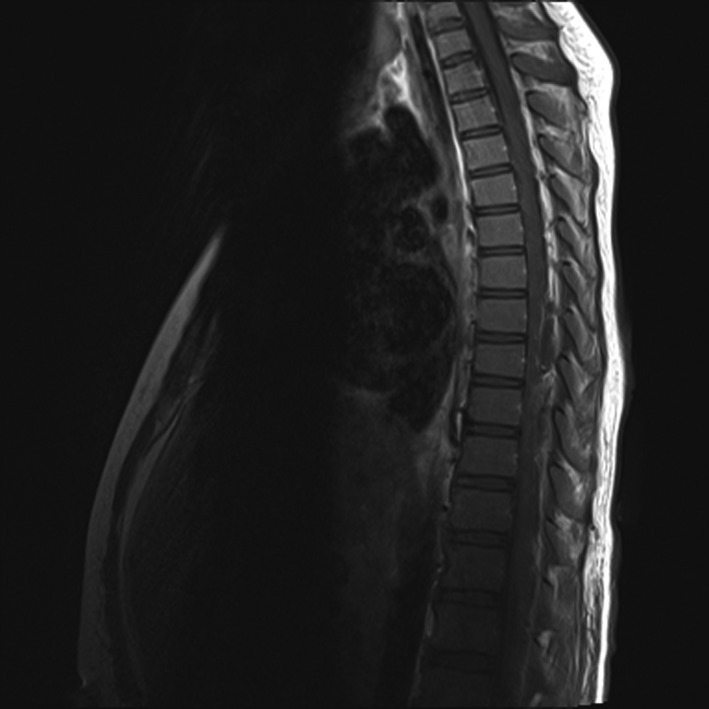
MRI T1 sagittal image is showing an iso‐intense lesion within the spinal canal spanning T7‐T10.

On the first postoperative day (POD) morning, the epidural infusion was stopped as he complained about the inability to move either leg. The catheter site was intact. Six hours later, he was still unable to move his right leg and had decreased sensation on both legs. On examination, the catheter was found to be dislodged and was thus removed. Emergent magnetic resonance imaging (MRI) showed a spinal EH at T7‐T9 with cord compression and hemoperitoneum (Fig.1, Fig.2 and Fig 3). Laboratory tests repeated at this time revealed Hgb 9.4 g∙dL^−1^, platelet count of 142 × 10^9^/L, INR of 1.44, aPTT of 36.9 sec, and elevated liver enzymes (ALT 1196 U/L, AST 3360 U/L). He underwent emergency spinal exploration approximately 20 h after the onset of his symptoms with the evacuation of 100 mL blood clot, T8 hemilaminectomy, and T7,T9 hemilaminotomy. Hemostasis was achieved with thrombin irrigation, bone wax, and bipolar cautery. Fresh frozen plasma (FFP) and Factor VII were given to treat coagulopathy. This was followed by exploratory laparotomy which revealed stenotic and edematous bile duct anastomosis, diffuse oozing of blood from the liver along the gall bladder bed, and 500 mL of blood clot evacuated from the abdomen. His recovery was complicated by sepsis, pleural effusion, acute kidney injury, and seizures. The workup for a bleeding diathesis was negative. Three weeks later, he had improved strength in his right leg and was discharged home on amlodipine, levetiracetam, and physical therapy. He had a complete recovery of strength at his 6‐month follow‐up visit.

## Discussion

This is a rare case of EH in a child with a thoracic epidural catheter. The bleeding complication was mainly attributed to coagulopathy caused by acute liver dysfunction. He was not on anticoagulant therapy. His workup for a congenital bleeding disease was negative. Sickle cell intrahepatic cholestasis (SCIC) characterized by extreme hyperbilirubinemia is a rare complication of SCD. It can result in acute liver synthetic dysfunction and coagulopathy. SCIC is usually managed by exchange transfusions 7. This patient had hyperbilirubinemia, anemia, normal liver enzymes, and coagulation profile a week before the procedure. He was responding to medical management with clinical improvement and decreasing bilirubin levels. On the day of the procedure, anemia was corrected, and the platelet count was normal. Coagulation testing was not repeated. In retrospect, routine coagulation testing on the day of the procedure would have been useful in ruling out coagulation defect before the surgical procedure. Postsurgery, he had an evolving liver injury with increasing bilirubin and liver enzymes and worsening coagulopathy. Bile drainage was obstructed by an edematous and stenotic anastomosis which was relieved by re‐exploration and placement of drainage tube. The INR reached a maximum value of 1.55 on POD 2. The coagulation laboratory values and the liver enzymes returned to his baseline by POD 7.

Uncomplicated use of a thoracic epidural catheter as part of the anesthetic management of a 35‐year‐old female patient with sickle cell disease undergoing common bile duct exploration has been reported 8. Although being a retrospective observational study and not related to the perioperative use of thoracic epidural anesthesia, epidural analgesia was safe and effective in treating pain crises in children with sickle cell disease 9. This case could have been managed with intravenous opioids for postoperative pain control. However, given the patient's history of acute chest syndrome and intermittent hypoxia requiring supplemental oxygen preoperatively, the risk/benefit discussions favored the use of an epidural catheter for optimal pain management.

We can only speculate the exact timing of EH formation. It could have happened at the time of insertion of the epidural catheter, or the dislodgement of the catheter may have played a role. His acute liver deterioration after the procedure, indicated by increasing liver enzymes and elevated INR, offers the most plausible cause for the bleeding complications.

In conclusion, this case report shows that practitioners should exercise caution in planning a neuraxial catheter technique in those patients who are at risk of deteriorating liver function in the perioperative period. Coagulation should be routinely tested before inserting a neuraxial catheter in this setting and closely monitored in the postoperative period. An epidural hematoma should be ruled out by radiological imaging in cases of a persistent motor block, especially in children who are preverbal or cannot cooperate with an examination.

## Ethics Approval

The patient's family reviewed the case report and gave written permission for the authors to publish the report.

## Authorship

Madhankumar Sathyamoorthy: participated in the case, wrote the original manuscript, edited and reviewed the final manuscript. Benjamin Walker: edited and reviewed the final manuscript. Melissa M. Rhodes^3^: edited and reviewed the final manuscript. Ike Eriator: edited and reviewed the final manuscript.

## Conflict of Interest

None declared.
